# Commentary: Sublingual Allergen Immunotherapy in HIV-Positive Patients

**DOI:** 10.3389/fimmu.2016.00132

**Published:** 2016-03-31

**Authors:** Salvatore Chirumbolo

**Affiliations:** ^1^Unit of Geriatry, Department of Medicine, University of Verona, Verona, Italy

**Keywords:** allergy and immunology, basophils, asthma, HIV, anti-retroviral therapy, immunotherapy, SLIT

I read with great interest the Brief Communication by Iemoli et al., on the latest issue of Allergy, where the authors reported that HIV-patients, undergoing anti-retroviral therapy (HAART) and sublingual immunotherapy (SLIT) to grass-pollen allergy, reported clinical benefits. The authors evaluated the outcome by the analysis of total combined score (TCS) and a quality of life (QoL) questionnaire (1) and concluded that SLIT was not only clinically effective but also safe and did not change any immune-virological parameter in HIV-infected individuals. Patients underwent immunotherapy while being in active HAART for at least 5 years and suffering from airway allergy even since many years before HIV infection. The authors limited their blood testing on CD4/CD8 ratio, CD4 amount, and HIV viral load. Previous reports have shown similar positive outcome, in SLIT or subcutaneous immunotherapy (2, 3).

The immunological status of a subject suffering from AIDS, though under retroviral therapy, deserves particular attention to her/his immune system, particularly about the role of the different CD4^+^ T-cell subpopulations. Actually, HIV-infected patients, undergoing HAART, are particularly susceptible to atopic predispositions and chronic allergy and, moreover, subjects immunization may enhance T-cell responses (3). Iemoli et al. reported that almost the whole cohort of individuals suffered from allergic rhinitis and 70% from asthma [(1), see Table 1].

The presence of allergic inflammation prior infection and the immune response of HIV-infected patients being under HAART may have a major role to diagnose and treat these individuals for pollen allergy (4). In this context, as Th-17 are strategic in allergy and may also contribute to the pathogenesis of classically recognized Th2-mediated allergic disorders, such as asthma, authors should be suggested to further investigate the role of Th-17 in these patients undergoing SLIT. During HIV infection, the role of Th17 may be impaired (5). In particular, Th-17 cells may be reduced in the gut of HIV-infected individuals, an occurrence that could lead to bacterial translocation from the gut lumen to the systemic circulation and chronic activation of the immune response (6). The paper by Iemoli et al. did not take into account the possible role exerted by these cells in mucosal immunity during oral immunotherapy in HIV-infected patients. From a pneumological point of view, the CD4^+^Th17 response, even in subjects with mild asthma due to pollen allergy, may activate CD177^+^ neutrophils, which can produce IL-17, accumulate in the lung, and exacerbate chronic allergy (7). This should occur in non-immune compromised patients. As during HIV infection, mucosal Th-17 function is altered and HAART is unable to restore the normalization of mucosal Th17 activity (8), the dysregulation of Th17 function might have a positive-driven role toward the resulted outcome from SLIT. Chronic inflammation during asthma is characterized by the fundamental role exerted by Th-17 and IL-23, which preferentially contributes in expanding Th-17, with neutrophil accumulation in airways and Th-2 cell-mediated basophil and eosinophil inflammation, thus exacerbating asthma symptoms (9). Iemoli and coworkers did not elucidate how much their reported outcome may depend on an impairment in mucosal immunity due to the Th-17/T-reg and Th-2 axis dysregulation.

Immune mucosal integrity is of major importance and serum IL-17 has even been related with allergy severity (10). The activity of Th-17 in these subjects is particularly puzzling, however. In HIV-infected patients, the role of CD4^+^Th17 is particularly important to elucidate the effect of immunization in these compromised individuals. In the article by Iemoli et al., HIV-infected subjects have CD4 T-cell absolute counts <500, a number comparable to that of the HIV discordant group reported in a recent paper by others, where patients reported a higher immune activation and immune exhaustion after being on HAART (11). Here, circulating Th17 T-cells paradoxically increased their number, compared with non-infected subjects (11). Effectiveness in SLIT in these patients may depend on the ability to restore CD4/CD8 ratio (concordant patients), on the relationship between regulatory T cells and Th-17 cells and on viral load being HAART. These parameters should be included when allergy immunization is performed in HIV-infected patients, as TCS may be used simply as an anamnestic index. Due to possible exacerbation of chronic immune response in these subjects, particularly following therapy, further evaluation of T-cell subtypes and particularly of γδ T-cells, might be fundamental to optimize serum immunotherapy against airway allergens and its follow up and/or maintenance. Even in HIV-infected patients, cytokine-secreting αβ^+^CD4^+^ T-helper 2 (Th2) cells orchestrate the type-2-driven immune response in a large proportion of atopic asthmatics. Th17 cells are present in bronchoalveolar lavage fluid of individuals with asthma. In HIV-infected patients, the role of Th17 and T-regs or the relationship Th17/T-reg/Th1 should be better focused, in order to elucidate if during immunotherapy mucosal barrier is compromised (Th17-depletion) or not or if allergy response is dampened (T-regs modulation) (12). The onset of an anergy-like state in the allergy response during HIV infection may be due by the initial increase in the T-reg/Th-17 ratio, possibly for Th-17 depletion, this may create some bias in the interpretation of the immunotherapy success. Usually, HIV-infected patients are characterized by depletion both in circulating Th17 and in CD161^+^ CD4^+^ T-cells, able to reconstitute Th17 pool, the presence of which is fundamental for the outcome of serum immunotherapy. This evidence raises some consideration if SLIT efficacy reported by the authors might account on a HAART-dependent recovery in mucosal Th17 activity or in an increased regulatory T cell-dependent dampening of allergy. The study from Iemoli et al. would gain major insights if blood specimens were analyzed for flow cytometry investigation of T-cell subtypes.

## Author Contributions

SC planned, conceived, wrote, discussed, and submitted the whole original manuscript.

## Conflict of Interest Statement

The author declares that the research was conducted in the absence of any commercial or financial relationships that could be construed as a potential conflict of interest.
